# Quantum-inspired squeeze-and-excitation in a time-conditioned U-Net for satellite image cloud removal

**DOI:** 10.3389/frai.2026.1808611

**Published:** 2026-06-29

**Authors:** Prithviraajan Senthilkumar, Hrishikesh Virupakshi, S. Kiruthika, J. Joshan Athanesious

**Affiliations:** School of Computer Science and Engineering, Vellore Institute of Technology, Chennai, India

**Keywords:** cloud removal, diffusion-based restoration, domain shift, hybrid quantum-classical learning, quantum-inspired attention, remote sensing, U-Net

## Abstract

Cloud occlusion severely restricts the use of optical satellite imagery for remote sensing applications, motivating the development of robust image restoration techniques. Current deep learning methods based on attention mechanisms and U-Net architectures have demonstrated superior results in cloud removal. However, these models rely on conventional feature recalibration techniques, which are insufficient to account for complex spatial correlations and long-range contextual dependencies. In this research, a hybrid quantum-classical image restoration framework for cloud removal in optical satellite data is proposed, which integrates Quantum Squeeze-and-Excitation (QSE) modules into a time-conditioned U-Net. The proposed model employs a diffusion-inspired noise-prediction objective, where sinusoidal timestep embeddings condition the network on varying noise levels, enabling stable and adaptive denoising. Classical squeeze-and-excitation blocks are replaced with parameterized quantum circuits that act as structured non-linear excitation functions, modeling inter-channel dependencies within a hybrid quantum-classical framework. The experimental study conducted on the RICE1 cloud removal dataset demonstrates that the QSE-enhanced U-Net consistently improves reconstruction quality compared to classical U-Net baselines in terms of peak signal-to-noise ratio (PSNR) and structural similarity index measure (SSIM) while maintaining stable training behavior. Additionally, tests were carried out on the EuroSAT dataset using artificially created Perlin noise-based cloud occlusions to assess cross-dataset transfer behavior. These findings provide a viable path for hybrid quantum-classical architectures in remote sensing image restoration by demonstrating that quantum-inspired channel attention can successfully improve feature recalibration in diffusion-style restoration networks.

## 1 Introduction

Optical satellite imagery (Yadav et al., 2024) plays a critical role in Earth observation applications such as environmental monitoring, agriculture, urban analysis, and disaster response due to its high spatial and temporal resolution. However, frequent and unpredictable cloud cover obscures surface information, reduces temporal continuity, and impairs the completion of downstream analytical tasks, thereby severely limiting the usefulness of optical images. It is estimated that more than half of spaceborne optical acquisitions are affected by cloud contamination, making reliable cloud removal a long-standing and fundamental challenge in remote sensing. The majority of early cloud removal techniques depended on manually developed heuristics and localized studies, which were frequently customized to specific regions or climatic circumstances and lacked generalizability. Large-scale, publicly accessible datasets made it possible to move toward data-driven and globally applicable solutions. A significant turning point in this change is the SEN12MS-CR dataset, which offers a combination of Sentinel-1 Synthetic Aperture Radar (SAR) and Sentinel-2 optical images across various geographic regions and seasonal circumstances. By providing a systematic assessment of learning-based approaches, SEN12MS-CR provided a framework for extensive cloud removal research. A widely used benchmark for cloud identification and restoration tasks, the RICE dataset (Lin et al., 2019), provides paired cloudy and cloud-free optical images.

By leveraging these datasets, deep learning-based image restoration techniques have made significant progress. Encoder-decoder architectures, particularly U-Net and its variations, have shown strong performance by simultaneously capturing both fine-grained local structures and broader contextual information. Subsequent improvements introduced gated convolutions (Dai et al., 2020), multi-scale feature fusion, and bi-temporal inputs to improve reconstruction consistency and explicitly differentiate between cloudy and clear regions. Attention mechanisms further improved these models by enabling adaptive emphasis on important spatial and channel-wise data (Zheng et al., 2024). For instance, it has been demonstrated that, particularly in thin cloud environments, channel attention approaches such as squeeze-and-excitation (SE) blocks (Hu et al., 2018) can effectively suppress cloud-related deviations while maintaining underlying land-cover patterns (Wen et al., 2022; Gu et al., 2019; Jiang et al., 2021).

Low-level image restoration research has evolved recently in response to generative modeling principles. Diffusion-based frameworks (Ho et al., 2020) have achieved state-of-the-art performance on a range of restoration tasks by incorporating recurrent denoising algorithmic methods that progressively transform damaged observations into clean reconstructions (Cui et al., 2024). Time-conditioned architectures (Ajith and Calhoun, 2025), which incorporate noise-level or timestep embeddings into the network, enable models to adaptively handle varying degrees of degradation and uncertainty. Such conditioning has proven particularly effective for restoration problems where corruption severity varies both spatially and temporally, including cloud-contaminated satellite imagery. Nevertheless, the majority of existing diffusion and time-conditioned restoration models rely exclusively on classical attention mechanisms for feature recalibration.

In parallel, advances in quantum computing and quantum-inspired machine learning have introduced complementary perspectives for representation learning (Le et al., 2011; Zhang et al., 2013; Alwan et al., 2025). Hybrid quantum-classical models and quantum neural networks have demonstrated enhanced expressibility and generalization capabilities (Abbas et al., 2021; Li et al., 2025), with successful applications in image classification (Song et al., 2024; Ng et al., 2024; Zollner et al., 2024), segmentation (Russo et al., 2025), and denoising (He et al., 2025; Kerger and Miyazaki, 2023). Among these advancements is the quantum squeeze-and-excitation (QSE) block (Peng et al., 2024), which allows for better modeling of inter-channel dependencies with compact parameterization by substituting parameterized quantum circuits for the fully connected layers of classical SE modules. Although QSE has demonstrated potential in high-level vision tasks, its potential advantages for low-level image restoration, especially in time-conditioned generative frameworks (Kölle et al., 2024), have not yet been investigated.

To discover innovative approaches for representational learning, optimization, and feature interaction, quantum machine learning (QML) has become a dynamic multidisciplinary research area that combines concepts of deep learning with quantum computing. The majority of QML techniques currently used in computer vision are primarily concerned with high-level recognition tasks, where quantum circuits are incorporated into classification pipelines to investigate non-linear feature transformations under compact parameterization.

On the other hand, relatively few studies have used quantum-inspired architectures for low-level image restoration challenges such as cloud removal, super-resolution, and denoising in conventional remote sensing data. These restoration challenges differ significantly from classification-oriented QML applications because they require precise reconstruction of spatial textures, structural continuity, and channel-wise feature dependencies.

By integrating variational quantum circuit-based channel recalibration into a diffusion-inspired optical cloud-removal architecture, the suggested QSE-enhanced Time-Conditioned U-Net establishes itself within this new class of hybrid quantum-classical restoration frameworks. This research aims to investigate whether channel dependence modeling during reconstruction may be enhanced by structured non-linear transformations and structured non-linear feature interactions generated by simulated variational quantum circuits. Additionally, rather than utilizing actual quantum hardware or Noisy Intermediate-Scale Quantum (NISQ) devices, all quantum operations are conducted via conventional simulations using the PennyLane framework. Instead of demonstrating quantum computing advantage or quantum supremacy, the proposed method should be considered a quantum-inspired hybrid learning framework.

This gap is particularly important for cloud removal from satellite images (Shubha et al., 2024; Painchart et al., 2024), where models need to be resilient to a variety of cloud topologies, different meteorological conditions, and domain transitions across geographical regions. Integrating quantum-inspired channel attention into temporally conditioned restoration architectures offers a promising direction for improving structural consistency and stability under various challenging conditions.

We present one of the first studies incorporating QSE blocks into a time-conditioned U-Net architecture for satellite image cloud removal to close this gap. The following is a summary of this study's primary contributions:

1. Novel architecture: to enable quantum-inspired channel recalibration within a time-conditioned image restoration framework, we propose a hybrid quantum-classical U-Net that substitutes QSE modules for classical SE blocks.
2. Systematic analysis: we evaluate hybrid QSE-SE setups against fully classical baselines and perform extensive ablation studies to examine the effects of QSE block placement within the encoder-decoder pipeline.
3. Empirical evaluation: to determine resilience under domain shift, we conduct cross-dataset transfer evaluation on noise-augmented EuroSAT imagery and test the proposed architecture on the RICE1 dataset (Lin et al., 2019).

Through these contributions, this study provides empirical evidence that quantum-inspired channel attention can enhance low-level satellite image restoration. Beyond demonstrating practical feasibility, the proposed approach establishes a foundation for future research on hybrid quantum-classical models for generative and time-conditioned remote sensing tasks.

## 2 Methodology

### 2.1 Problem formulation

Let **x**_**0**_ denote a clean optical satellite image and **x**_**c**_ its corresponding cloud-contaminated observation. The objective of cloud removal is to learn a function **f**_**θ**_ that reconstructs **x**_**0**_ from **x**_**c**_. This task is treated as a conditional image restoration problem under a diffusion-inspired noise formulation.

### 2.2 Overall framework

**Figure 1:**
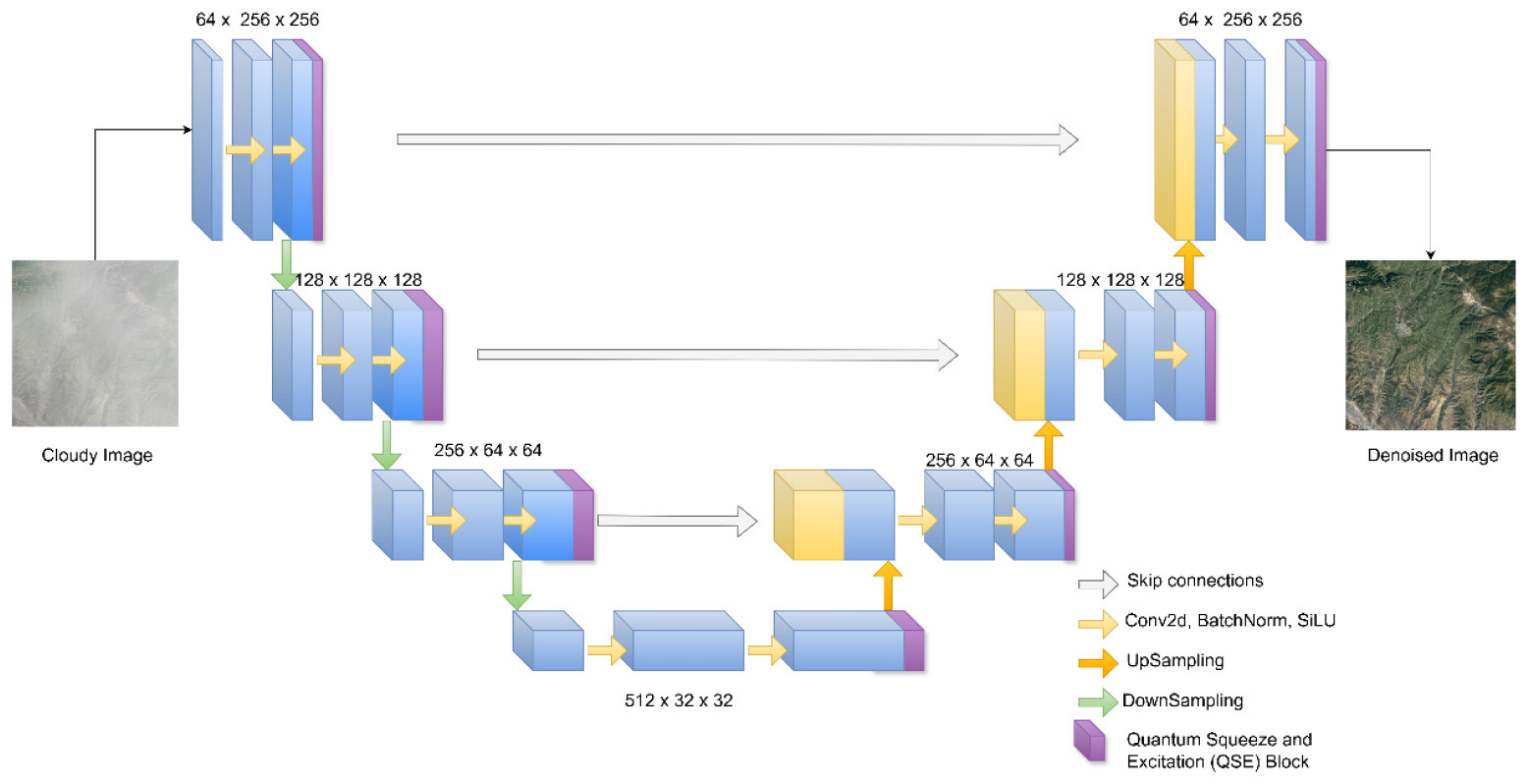
Proposed time-conditioned U-Net architecture with integrated QSE modules. QSE, quantum squeeze-and-excitation.

The proposed approach employs a time-conditioned U-Net architecture augmented with QSE modules. The network is trained to predict the noise residual between cloudy and clean images at varying noise levels. Temporal conditioning is achieved using sinusoidal timestep embeddings (Ho et al., 2020), allowing the model to adapt its denoising behavior across different corruption strengths. Classical squeeze-and-excitation blocks (Hu et al., 2018) are replaced with quantum-inspired attention modules that use parameterized quantum circuits (Abbas et al., 2021; Li et al., 2025) to perform channel recalibration. The resulting framework forms a hybrid quantum-classical restoration model that combines spatial feature extraction, temporal noise awareness, and quantum-inspired channel attention. Figure 1 provides an overview of the suggested time-conditioned U-Net with integrated QSE modules.

### 2.3 Dataset and preprocessing

**Figure 2:**
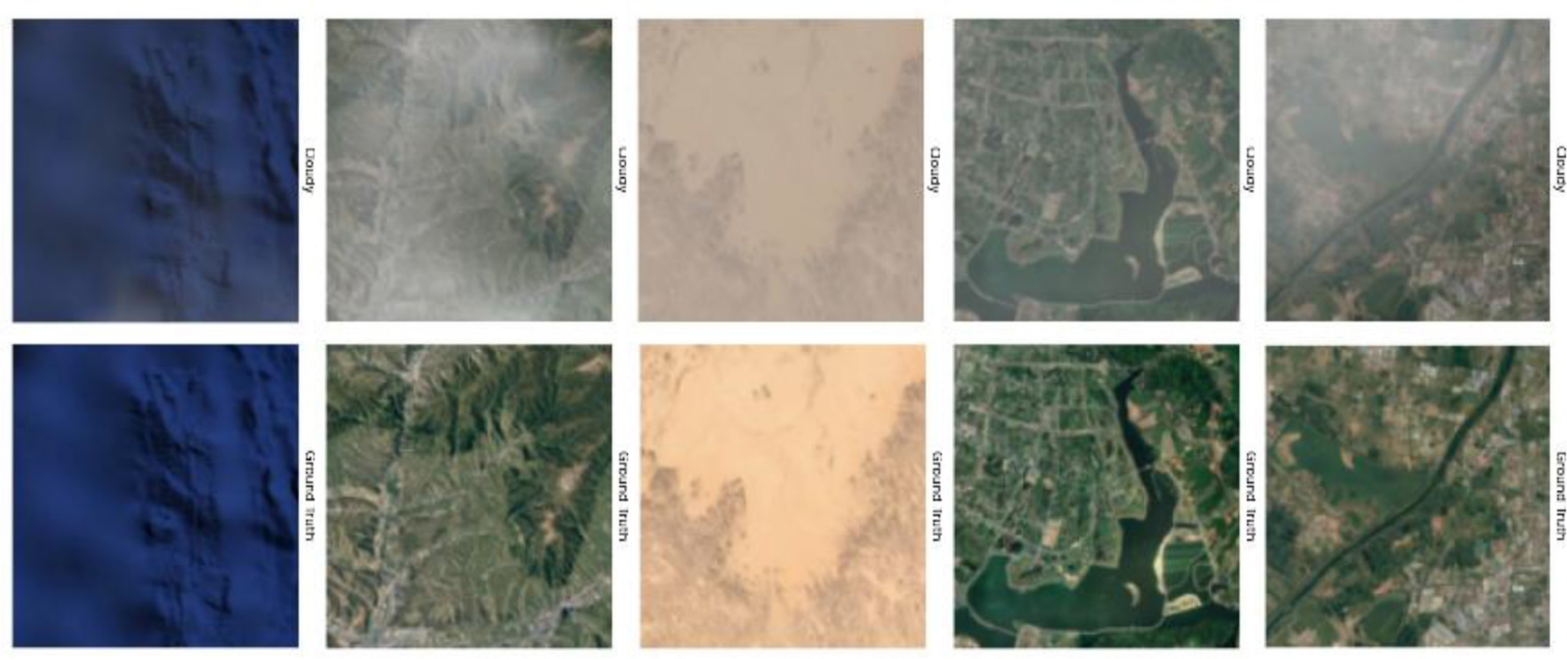
Sample images from the RICE1 dataset, with the cloudy images on top and the cloud-free ground truth on the bottom.

Paired cloudy and cloud-free optical remote sensing images developed especially for supervised cloud-removal research make up the RICE1 dataset (Lin et al., 2019). Direct pixel-wise reconstruction assessment with paired supervision becomes possible through the dataset's 500 pairs of naturally cloud-contaminated optical scenes and their matching cloud-free references. The proposed framework may be evaluated across diverse spatial structures and scene characteristics, including urban regions, vegetation, agricultural areas, and water bodies under various atmospheric circumstances. To ensure consistent optimization behavior while preserving the structural content needed for cloud-removal evaluation, all images used in this study were scaled to a predefined spatial resolution of 128 × 128 and normalized to the [0,1] range before training. No explicit cloud masks are used, and cloud removal is learned implicitly through reconstruction from paired data, as shown in Figure 2.

For cross-dataset evaluation, the model pretrained on RICE1 is further fine-tuned on synthetically cloud-degraded EuroSAT images. This setting reflects a practical domain adaptation scenario, enabling analysis of cross-dataset transfer behavior under domain shift. Since EuroSAT does not contain naturally paired cloudy and cloud-free images, synthetic cloud-like degradations are generated using Perlin noise patterns. The Perlin noise is applied via global alpha blending, producing spatially correlated, thin atmospheric haze distributed across the entire image to simulate diffuse cloud coverage rather than localized occlusions. The generated noise is applied globally across the image as a thin semi-transparent layer, creating controlled cloud-corrupted inputs, while the original clean images serve as ground truth targets.

We highlight that this research does not consider SAR-optical datasets such as SEN12MS-CR (Ebel et al., 2022), which often show temporal inconsistencies between cloudy and cloud-free acquisitions because of atmospheric variability, seasonal changes, illumination variations, and land-cover evolution over acquisition times. Perfectly aligned paired supervision is challenging for controlled cloud-removal assessment because of these considerations. On the other hand, the RICE1 dataset offers paired cloudy and cloud-free optical images developed especially for guided cloud-removal benchmarking, enabling a controlled assessment of structure preservation and reconstruction accuracy under uniform optical acquisition conditions. Furthermore, the current scope of this study focuses on purely optical cloud removal using paired supervision rather than cross-modal fusion. Extending the framework to SEN12MS-CR constitutes an important direction for future investigation.

Considering that the original training dataset (RICE1) and the target domain (EuroSAT) are clearly distinct, this controlled fine-tuning approach enables evaluation of the proposed model's adaptability to a substantially different dataset.

### 2.4 Time-conditioned U-net architecture

A U-Net-style encoder-decoder architecture with symmetric skip links between corresponding resolution levels serves as the foundation for the suggested restoration network. Let the input cloudy image be denoted as $x_{c}\in {\mathbb{R}}^{H\times W\times 3}$. To enable hierarchical feature extraction from local textures to global scene structure, the encoder gradually decreases spatial resolution while increasing channel dimensionality. The feature transformation at each encoder step *l* is given in Equation 1:


$$\begin{array}{l} {\text{h}}_{l}=\phi \left({\text{Conv}}_{l}\left({\text{h}}_{l-1}\right)+\psi \left(t\right)\right) \end{array}$$ (1)

where h_*l*−1_ is the input feature map, Conv_*l*_(·) denotes a convolutional block, ψ(*t*) represents the projected timestep embedding, and ϕ(·) is a non-linear activation function. The addition of ψ(*t*) conditions the feature activations on the noise level.

The decoder mirrors the encoder by gradually restoring spatial resolution through upsampling operations. Fine-grained spatial details are preserved by concatenating encoder and decoder features at corresponding resolution levels via skip connections. Cloud removal in high-resolution satellite imagery depends on the model's ability to integrate localized reconstruction cues with global contextual understanding.

### 2.5 Sinusoidal timestep embedding

To enable noise-level awareness, the model incorporates explicit time conditioning using sinusoidal timestep embeddings. For a given timestep *t*∈{1, …, *T*}, a continuous embedding vector $e_{t}\in {\mathbb{R}}^{d}$ is constructed as shown in Equation 2:


$$\begin{array}{l} {\text{e}}_{t}^{\left(2i\right)}=sin\left(t/{10,000}^{2i/d}\right),{\text{e}}_{t}^{\left(2i+1\right)}=\cos\left(t/{10,000}^{2i/d}\right) \end{array}$$ (2)

where *i* indexes the embedding dimensions and *d* is the embedding size.

The resulting embedding is passed through a small multilayer perceptron to match the feature dimensionality of each U-Net block, as shown in Equation 3:


$$\begin{array}{l} \psi \left(t\right)=W_{2}\sigma \left(W_{1}e_{t}\right) \end{array}$$ (3)

where *W*_1_ and *W*_2_ are learnable linear projections and σ(·) denotes the SiLU activation. The projected embedding is then added to intermediate feature maps, enabling the network to adapt its denoising behavior continuously across different noise intensities.

### 2.6 Quantum squeeze-and-excitation module

The QSE module employed in this study was originally introduced in a prior conference publication (Peng et al., 2024) and is adopted here as a building block within a diffusion-style image restoration framework. In this design, the QSE module replaces the classical excitation mechanism used for channel attention (Hu et al., 2018) by leveraging parameterized quantum circuits (Abbas et al., 2021; Li et al., 2025) for channel-wise feature recalibration. All quantum components in this study are implemented using differentiable quantum circuit simulators on classical hardware and should therefore be interpreted as quantum-inspired hybrid learning mechanisms rather than as deployments on physical NISQ quantum devices. Given an intermediate feature tensor *F*∈ℝ^*B*×*C*×*H*×*W*^, where B, C, H, and W denote the batch size, number of channels, height, and width, respectively, global average pooling is first applied to obtain a channel descriptor, as shown in Equation 4:


$$\begin{array}{l} z_{c}=\frac{1}{HW}\overset{H}{\underset{i=1}{\sum }}\overset{W}{\underset{j=1}{\sum }}{\text{F}}_{c,i,j} \end{array}$$ (4)

The resulting vector **z**∈ℝ^*C*^ is encoded into a quantum state ∣ψ_0_〉 using amplitude encoding (Zhang et al., 2013; Alwan et al., 2025), using amplitude encoding shown in Equation 5:


$$|\psi_{0}\rangle =\sum_{c=1}^{C}z_{c}|c\rangle$$ (5)

where ∣*c*〉 denotes the computational basis state corresponding to the *c*-th channel. This quantum state is processed by a parameterized variational quantum circuit composed of rotation gates and entangling operations (Abbas et al., 2021; Song et al., 2024), yielding the output shown in Equation 6:


$$|\psi (\Theta )\rangle =U(\Theta )|\psi_{0}\rangle$$ (6)

where *U*(Θ) is a unitary operator parameterized by trainable parameters Θ. Measurement of Pauli-*Z* observables yields expectation values, as shown in Equation 7:


$$\begin{array}{l} q_{c}=\langle \psi \left(\Theta \right)|Z_{c}|\psi \left(\Theta \right)\rangle \end{array}$$ (7)

where *Z*_*c*_ is the Pauli-*Z* operator acting on the *c*-th qubit, and *q*_*c*_ represents the corresponding quantum feature. These quantum features are projected back to channel weights through a learnable linear transformation followed by a sigmoid activation, as shown in Equation 8:


$$\begin{array}{l} s=\sigma \left(W_{q}q\right) \end{array}$$ (8)

where *W*_*q*_ is a trainable weight matrix and σ(·) denotes the sigmoid function. The original feature maps are recalibrated shown in Equation 9


$$\begin{array}{l} {\tilde{\text{F}}}_{c}=s_{c}\cdot {\text{F}}_{c} \end{array}$$ (9)

The forward pass of the QSE module is summarized as follows:

1. Input feature tensor *F*∈ℝ^*B*×*C*×*H*×*W*^.
2. Global average pooling is used to obtain the channel descriptor *z*∈ ℝ^*C*^.
3. Amplitude encoding is used to encode *z* into a quantum state.
4. Pass the encoded state through the variational quantum circuit *U*(Θ).
5. Quantum features (*q*) are obtained using the Pauli-*Z* expectation.
6. Apply linear projection and sigmoid activation to generate channel weights (*s*).
7. Recalibrate feature maps using ${\tilde{F}}_{c}=s_{c}\cdot F_{c}$.
8. Output the recalibrated feature tensor $\tilde{F}$.

**Figure 3:**
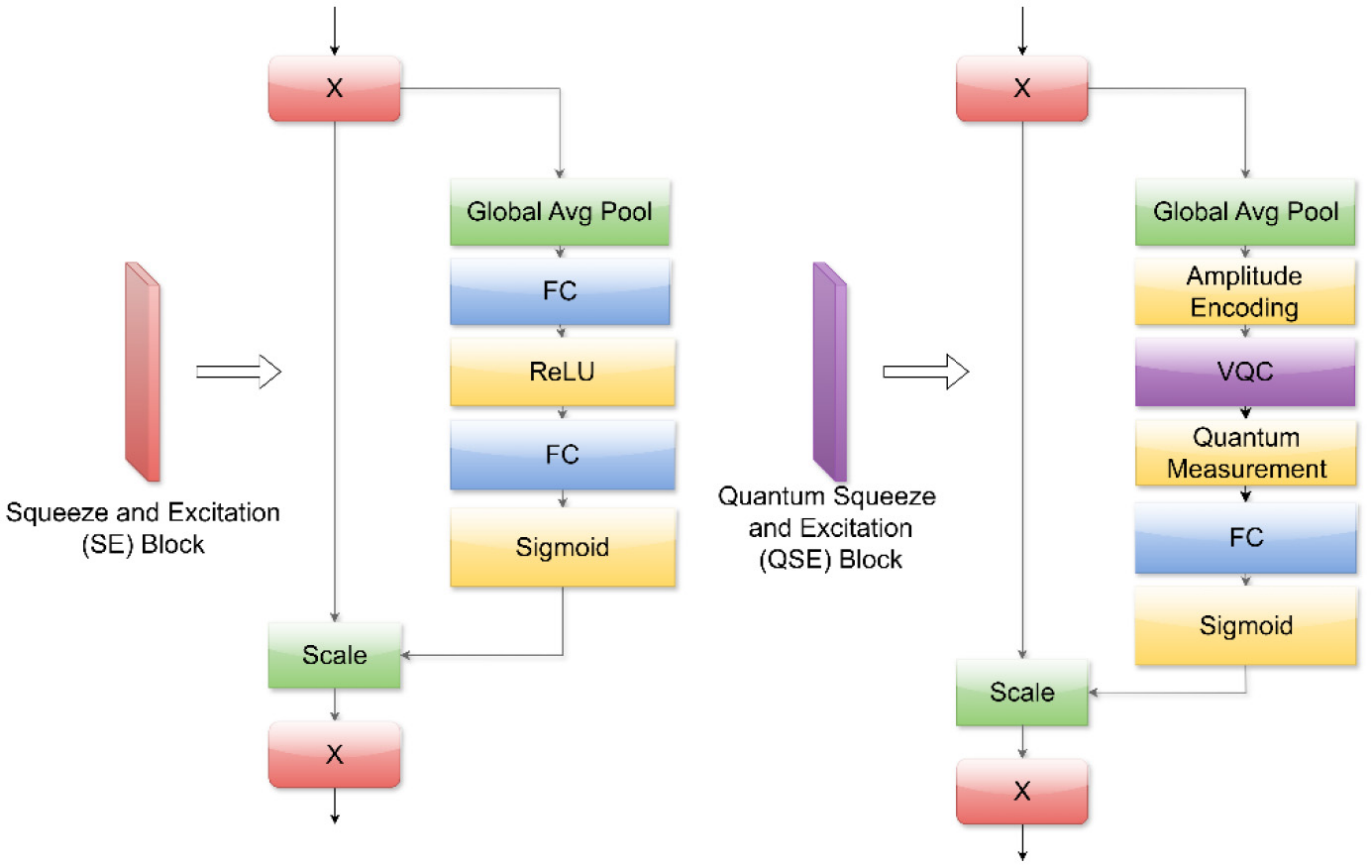
Comparison between classical SE and QSE. QSE, quantum squeeze-and-excitation; SE, squeeze-and-excitation.

Figure 3 illustrates the conceptual difference between the classical squeeze-and-excitation mechanism and the proposed quantum-inspired squeeze-and-excitation module. This hybrid attention mechanism enables structured non-linear interactions between channels that are difficult to model using classical fully connected layers alone. Table 1 summarizes the structure and components of the QSE module. In contrast to the original formulation, the present study focuses on the integration, architectural placement, and empirical impact of QSE within a time-conditioned U-Net for cloud removal in optical satellite imagery.

**Table 1:** Structure and components of the QSE module, including quantum encoding, circuit configuration, and output mapping.

Component	Description
Input	Channel-wise pooled feature vector
Pooling	Global average pooling
Encoding	Amplitude encoding
Number of qubits	log_2_(*C*)
Variational layers	2
Parameterized gates	RY rotations
Entanglement	Nearest-neighbor CNOT
Measurement	Pauli-*Z* expectation values
Output mapping	Linear projection + sigmoid
Integration	Channel-wise feature recalibration

CNOT, controlled-NOT; QSE, quantum squeeze-and-excitation; RY, rotation about the Y-axis.

### 2.7 Diffusion-inspired training objective

Training follows a simplified diffusion-based noise-prediction objective. Let *x*_0_ denote a clean image and ϵ~𝒩(0, *I*) Gaussian noise. A noisy image at timestep (*t*) is generated as shown in Equation 10:


$$\begin{array}{l} x_{t}=\sqrt{\alpha_{t}}x_{0}+\sqrt{1-\alpha_{t}}\epsilon \end{array}$$ (10)

where α_*t*_ is defined by a predefined noise schedule. The model ϵ_θ_(·) is trained to predict the noise component given the noisy image and timestep, as shown in Equation 11:


$$\begin{array}{l} \hat{\epsilon }=\epsilon_{\theta }\left(x_{t},t\right) \end{array}$$ (11)

The optimization objective minimizes the mean squared error between predicted and true noise (ℒ_*diff*_), as shown in Equation 12:


$$\begin{array}{l} L_{diff}=E_{x_{0},\epsilon ,t}\left[∥\epsilon -\epsilon_{\theta }\left(x_{t},t\right){∥}_{2}^{2}\right] \end{array}$$ (12)

This formulation stabilizes training and allows the network to generalize across varying noise levels without explicitly modeling the full reverse diffusion process. Rather than providing a comprehensive denoising diffusion probabilistic model using iterative reverse sampling, the proposed framework should be understood as a diffusion-inspired time-conditioned regression model. The primary goals of the diffusion-style approach are to stabilize restoration across different deterioration levels and implement timestep-aware noise conditioning.

### 2.8 Optimization and implementation details

All network parameters, including quantum circuit parameters, are optimized jointly using the Adam optimizer. Training is performed using mini-batch stochastic gradient descent with early stopping based on validation PSNR and SSIM. Quantum circuits are simulated using a differentiable quantum framework, enabling gradient backpropagation through expectation-value measurements.

### 2.9 Ablation strategy

To isolate the impact of quantum-inspired attention, ablation studies are conducted by varying the placement of QSE modules within the encoder, decoder, and bottleneck layers. Additional experiments vary the depth of the variational quantum circuit. Performance is evaluated using PSNR and SSIM metrics to quantify reconstruction fidelity and structural preservation. Results of the ablations are presented in the following section.

This methodology introduces a hybrid quantum-classical image restoration framework that integrates quantum-inspired channel attention into a time-conditioned U-Net trained using a diffusion-style objective. The proposed design enables noise-adaptive denoising, enhanced channel dependency modeling, and stable end-to-end optimization, making it suitable for cloud removal in optical satellite imagery.

## 3 Experimental setup, analysis, and discussion

### 3.1 Datasets

All experiments are conducted using the RICE1 dataset (Lin et al., 2019), which provides paired cloudy and cloud-free optical satellite images for supervised cloud removal. The dataset is split into training and validation subsets with no overlap between samples. All images are resized to a fixed spatial resolution of 128 × 128 pixels and normalized to the [0, 1] intensity range before model training. To evaluate cross-dataset transfer behavior under distribution shift, additional experiments are performed on the EuroSAT dataset. Since EuroSAT does not include cloud-paired ground truth, synthetic cloud-like occlusions are generated using Perlin noise patterns. These synthetically corrupted images are used for controlled fine-tuning and evaluation to analyze cross-dataset transfer behavior under domain shift.

### 3.2 Implementation details

All experiments use the model architecture described in Section 3. An NVIDIA GeForce RTX 3060 GPU with CUDA 11.8 support, an Intel Core i7 (11th generation) processor, and 64 GB of RAM were used to test the suggested framework, which was implemented in PyTorch with quantum-inspired components realized using PennyLane. During validation, reconstructed outputs are regularly saved to visualize qualitative findings. Quantum circuits are simulated using a differentiable qubit simulator and integrated into the PyTorch computational graph, enabling end-to-end optimization without reliance on physical quantum hardware. Training is performed using mini-batch stochastic gradient descent with a batch size of 32. The Adam optimizer is employed with a fixed learning rate of 1 × 10^−4^. No learning-rate scheduling is applied. The complete set of training and model hyperparameters is reported in Table 2. Table 3 illustrates the layer-wise breakdown of the proposed time-conditioned U-Net architecture, including feature dimensions and activation functions.

**Table 2:** Training configuration and hyperparameters used for all experiments.

Category	Parameter	Value
Input	Image resolution	128 × 128
Input	Input channels	3 (RGB)
Diffusion	Number of timesteps (*T*)	1,000
Diffusion	Noise prediction target	
Network	Base feature dimension	64
Network	Encoder depth	Three stages
Network	Bottleneck channels	512
Attention	QSE circuit depth	Two layers
Attention	Quantum encoding	Amplitude encoding
Attention	Measurement	Pauli-*Z* expectation
Optimization	Optimizer	Adam
Optimization	Learning rate	1 × 10^−4^
Optimization	Batch size	32
Optimization	Early stopping	Yes (patience = 5)
Evaluation	Metrics	PSNR, SSIM
Hardware	GPU	NVIDIA RTX 3060
Quantum backend	Simulator	PennyLane default qubit

PSNR, peak signal-to-noise ratio; QSE, quantum squeeze-and-excitation; RGB, red-green-blue; SSIM, structural similarity index measure.

**Table 3:** Layer-wise breakdown of the proposed time-conditioned U-Net architecture, including feature dimensions and activation functions.

Stage	Block name	Input shape	Output shape	Attention	Activation
Encoder	DownBlock-1	128 × 128 × 3	128 × 128 × 64	QSE	SiLU
Encoder	DownBlock-2	64 × 64 × 64	64 × 64 × 128	QSE	SiLU
Encoder	DownBlock-3	32 × 32 × 128	32 × 32 × 256	QSE	SiLU
Bottleneck	Bottleneck Block	16 × 16 × 256	16 × 16 × 512	QSE	SiLU
Decoder	UpBlock-1	16 × 16 × 768	32 × 32 × 256	QSE	SiLU
Decoder	UpBlock-2	32 × 32 × 384	64 × 64 × 128	QSE	SiLU
Decoder	UpBlock-3	64 × 64 × 192	128 × 128 × 64	QSE	SiLU
Output	Final Conv	128 × 128 × 64	128 × 128 × 3	None	Linear

QSE, quantum squeeze-and-excitation; SiLU, sigmoid linear unit.

### 3.3 Training protocol

Training follows a diffusion-inspired noise-prediction framework (Ho et al., 2020) with a total of *T* = 1000 timesteps. At each iteration, a timestep is randomly sampled and the network is trained to predict the residual noise between cloudy and clean images using a mean squared error objective. To prevent overfitting and ensure stable convergence, early stopping is applied based on validation performance with a patience of five epochs. Training is terminated when no improvement in validation PSNR is observed over this window. The model checkpoint achieving the highest validation PSNR is retained for subsequent evaluation.

Reconstruction quality is evaluated using PSNR and SSIM. Denoised outputs, which have been clipped to the permissible intensity range, are employed to generate metrics. While SSIM provides an additional assessment of structural faithfulness, PSNR is the primary benchmark for model selection. Both quantitative and qualitative analyses are used to evaluate reconstruction quality, training stability, and the impact of quantum-inspired channel attention. After fine-tuning, cross-dataset transfer behavior is evaluated using the EuroSAT dataset. The RICE1 dataset (Lin et al., 2019) is employed for primary evaluation, and the findings are then compared with both classical baselines and internal ablation variations. The primary measures used to assess performance are PSNR and SSIM.

### 3.4 Computational complexity and inference analysis

We examined the computational costs of the conventional SE-UNet and the proposed QSE-Decoder architecture in terms of training time, inference latency, and parameter count to determine the feasibility of the hybrid quantum-classical framework. Using 128 × 128 RGB satellite image patches, all experiments were carried out under the same training circumstances. Due to the additional computational overhead generated by variational quantum circuit (VQC) simulation, the QSE-Decoder model required approximately 1.2 min per epoch, while SE-UNet achieved an average training time of around 0.25 min per epoch (Table 4). In a similar vein, the QSE-Decoder model's inference latency increased from around 5.72 ms/image for SE-UNet to approximately 55.54 ms/image.

**Table 4:** Computational complexity comparison between SE-UNet and QSE-Decoder architectures.

Model	Parameters	Training time/epoch	Inference time/image
SE-UNet	7,935,963	~0.25 min	~5.72 ms
QSE-Decoder	7,928,553	~1.20 min	~55.54 ms

QSE, quantum squeeze-and-excitation; SE, squeeze-and-excitation; UNet, U-Net.

The QSE-based architecture maintained a parameter count comparable to that of the classical baseline despite the extended runtime, indicating that the enhanced feature recalibration mechanism produced by the quantum-inspired excitation module was the primary driver of the performance gains rather than parameter scaling. Amplitude-embedding procedures and repetitive quantum circuit evaluations carried out throughout training and inference were the main drivers of the increased computing expense.

The observed overhead remains acceptable for offline satellite image restoration workflows and experimental remote sensing applications, where reconstruction quality is often prioritized over strict real-time constraints.

### 3.5 Quantitative comparison with existing models

The proposed method and the reported performance of current cloud-removal techniques on the RICE1 dataset are compiled in Table 5. The results for earlier approaches are presented for contextual comparison and are taken directly from the corresponding publications.

**Table 5:** Quantitative comparison of cloud-removal performance on the RICE1 dataset using PSNR and SSIM.

Model [ref]	PSNR (dB)	SSIM
IDeRS (Xu et al., 2019)	14.28	0.5324
DCP (Zhang et al., 2018)	20.563	0.8927
SpA-GAN (Pan, 2020)	29.749	0.9541
SE-UNet (prev work)	29.87	0.9582
**QSE-UNet (proposed)**	**30.35**	**0.9629**

Results for existing methods are reported from their respective publications.

DCP, dark channel prior; PSNR, peak signal-to-noise ratio; QSE, quantum squeeze-and-excitation; SE, squeeze-and-excitation; SSIM, structural similarity index measure. Bold values represent the best results for the corresponding metric.

Due to their reliance on manually created priors and the absence of adaptive feature modeling, conventional techniques such as IDeRS (Xu et al., 2019) and Dark Channel Prior (Zhang et al., 2018) show poor reconstruction capabilities. Although generative adversarial network (GAN)-based techniques such as SpA-GAN (Pan, 2020) show better perceptual quality, they are sensitive to adversarial optimization and exhibit unstable training. When compared to the classical U-Net and SE-U-Net baselines, the suggested QSE-enhanced U-Net delivers better PSNR and SSIM. Quantum-inspired channel attention leads to a quantifiable improvement in both reconstruction fidelity and structural preservation when compared to the SE-U-Net. These improvements show that, even when trained under the same diffusion-style objectives (Ho et al., 2020), QSE modules offer more expressive channel recalibration than classical excitation mechanisms (Hu et al., 2018). Since variational quantum circuits can project channel descriptors into a higher-dimensional Hilbert-space-inspired representation, complex non-linear inter-channel interactions can be modeled within a compact parameterization regime, resulting in enhanced expressivity. The benefit of the QSE module is not claimed to arise from quantum supremacy. Instead, the VQC acts as a structured non-linear transformation that introduces entangled channel interactions under a compact parameterization regime. The observed gains likely arise from the ability of the variational circuit to model higher-order channel correlations using fewer trainable parameters rather than from any demonstrated quantum computational advantage.

Diffusion-based restoration approaches and SAR-optical fusion datasets, such as SEN12MS-CR, where additional SAR imagery provides supplementary structural information under dense cloud cover, have become increasingly important components of modern state-of-the-art cloud removal frameworks. On the other hand, the present study utilizes paired optical supervision derived from the RICE1 dataset to specifically focus on purely optical cloud removal. In addition, the proposed approach uses diffusion-inspired time-conditioned noise prediction within a lightweight restoration framework, in contrast to full denoising diffusion probabilistic models that use iterative reverse sampling.

### 3.6 Statistical significance analysis

To further evaluate the consistency and stability of the proposed framework, paired statistical analysis was conducted using per-image restoration metrics obtained from the evaluation set. A paired *t*-test was performed between the SE-UNet baseline and the proposed QSE-UNet architecture to examine whether the observed performance differences could be attributed to the proposed quantum-inspired feature recalibration mechanism rather than random initialization effects or architectural noise.

The paired statistical analysis yielded *P*-values of 0.6523 and 0.8925 for the evaluated restoration metrics. Although the observed quantitative improvements were modest, the proposed QSE-Decoder architecture consistently demonstrated competitive reconstruction performance while maintaining stable convergence behavior across training epochs. These observations suggest that the integration of VQC-based excitation modules contributes to effective channel-wise feature recalibration while preserving reconstruction stability and parameter efficiency within the proposed hybrid quantum-classical framework.

### 3.7 Internal baseline comparison

The proposed framework is contrasted with a number of internally implemented baselines that were trained under the same circumstances to isolate the impact of quantum-inspired attention. These consist of a traditional SE-U-Net, a residual U-Net, and a standard U-Net (Hu et al., 2018; Gu et al., 2019; Jiang et al., 2021). The comparison between the suggested model and its internal baselines is compiled in Table 6.

**Table 6:** Comparison of reconstruction performance between the proposed model and internally implemented baseline architectures trained under identical settings.

Model	PSNR (dB)	SSIM
U-Net	25.87	0.9324
Residual U-Net	24.84	0.9546
SE**-**UNet	29.87	0.9582
**QSE-UNet (proposed)**	**30.35**	**0.9629**

PSNR, peak signal-to-noise ratio; QSE, Quantum Squeeze-and-Excitation; SE, Squeeze-and-Excitation; SSIM, structural similarity index measure. Bold values represent the best results for the corresponding metric.

The QSE-U-Net provided improved performance relative to all internal baselines in terms of PSNR and SSIM on the RICE1 dataset, indicating the efficacy of quantum-inspired channel attention. After fine-tuning, QSE-based decoding produces higher SSIM with lower variation than our previous SE-All architecture during cross-dataset transfer to EuroSAT, suggesting better structure preservation, while SE-All achieves a slightly greater PSNR. When compared to the conventional SE-U-Net on RICE1, the QSE-enhanced variant exhibits better structural coherence and fewer cloud artifacts, yielding modest but consistent PSNR and SSIM improvements. Crucially, these gains are achieved without significantly increasing the overall number of trainable parameters, indicating that better feature recalibration, rather than greater model capacity, is the source of the performance gain.

### 3.8 Effect of QSE placement

An ablation experiment was conducted to investigate the impact of QSE block placement in the U-Net design. QSE modules were selectively inserted into the encoder, bottleneck, decoder, or all stages of the network, while classical SE blocks (Hu et al., 2018) were retained elsewhere. The quantitative results of the analysis are summarized in Table 7.

**Table 7:** Effect of QSE module placement within the U-Net architecture on reconstruction performance, convergence behavior, and quantum resource usage.

QSE placement	PSNR	SSIM	Loss	Best Epoch	Param	Qubits
ENCODER	28.01	0.9138	0.0022	17	7,900,379	21
DECODER	**30.35**	**0.9529**	**0.0016**	31	7,928,553	21
BOTTLENECK	24.81	0.9292	0.0024	20	7,907,789	9
ALL	26.15	0.93	0.0022	17	7,892,969	51

PSNR, peak signal-to-noise ratio; QSE, quantum squeeze-and-excitation; SSIM, structural similarity index measure. old values represent the best results for the corresponding metric.

Out of all configurations, decoder-only QSE integration yields the best reconstruction performance, with the highest PSNR and SSIM. This result suggests that quantum-inspired channel attention is most effective during the reconstruction phase, when high-frequency features and spatial consistency are recovered. As circuit depth increases, more non-linear transformations and entanglement operations are implemented, which might cause optimization to become unreliable and increase gradient sensitivity during training. Excessive non-linear transformations may overmix intermediate channel representations and attenuate high-frequency spatial information necessary for accurate restoration. Encoder-only QSE integration provides a moderate improvement, whereas bottleneck-only placement yields negligible benefits, possibly due to reduced feature diversity at highly compressed representations. Applying QSE blocks at every level does not improve performance; in fact, it slightly degrades it, despite utilizing more quantum resources. Excessive insertion of QSE modules may repeatedly recalibrate intermediate representations, leading to unstable feature transformations and more difficult gradient propagation. Since each variational quantum circuit introduces additional non-linear transformations and entanglement operations, stacking QSE modules at every level may amplify optimization sensitivity and reduce convergence stability. This implies that redundant transformations brought about by excessive quantum recalibration may hinder optimization. All things considered, these findings demonstrate that achieving optimal performance requires strategic placement of QSE modules.

### 3.9 Effect of quantum circuit depth

The effect of quantum circuit complexity was investigated through experiments with different quantum layers in the QSE module (Li et al., 2025; Song et al., 2024). The quantitative results of the analysis are summarized in Table 8. The limited reconstruction capabilities of a shallow design with a single quantum layer indicate inadequate expressiveness. Increasing the circuit depth to two layers yields the best overall performance and consistent convergence, greatly enhancing both PSNR and SSIM. When the circuit depth is increased to four or six layers, performance suffers. These deeper configurations exhibit worse stability and higher reconstruction loss, most likely due to over-parameterization and more difficult quantum circuit optimization. The degradation observed for deeper variational circuits may also be associated with barren plateau effects (Larocca et al., 2025) commonly reported in variational quantum optimization, where gradients become increasingly flat as circuit depth increases. These results imply that the optimal balance between training stability and representational power is provided by a modest quantum depth.

**Table 8:** Impact of quantum circuit depth within the QSE module on reconstruction quality and training stability.

n_layers	PSNR	SSIM	Loss	Best epoch
1	22.4	0.8865	0.0091	6
2	**30.35**	**0.9529**	**0.0016**	31
4	24.59	0.897	0.0041	11
6	25.66	0.9167	0.003	14

PSNR, peak signal-to-noise ratio; QSE, Quantum Squeeze-and-Excitation; SSIM, structural similarity index measure. Bold values represent the best results for the corresponding metric.

### 3.10 Qualitative analysis

**Figure 4:**
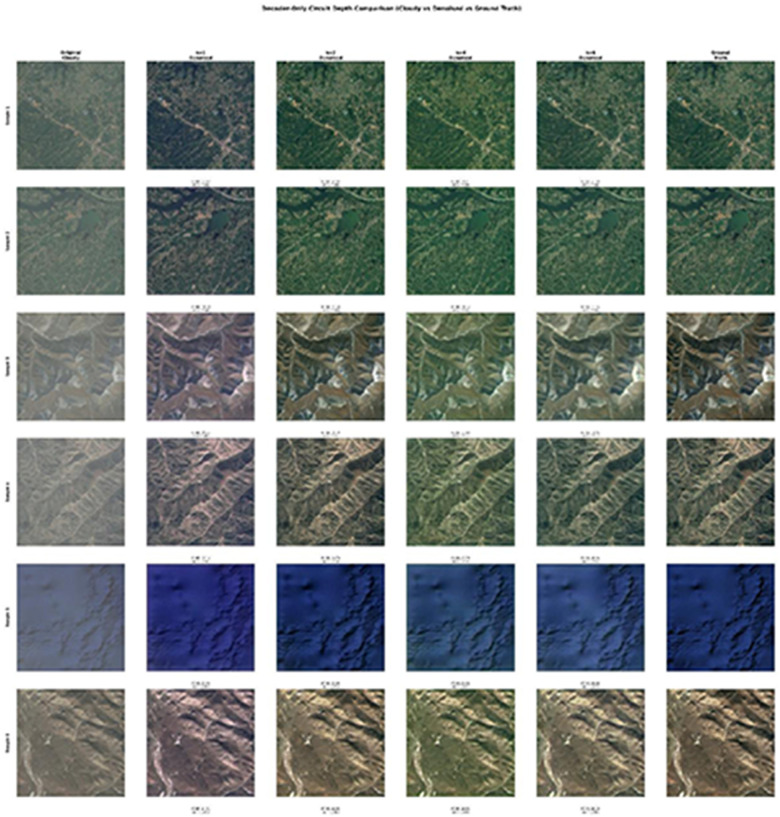
Qualitative cloud-removal results for varying quantum circuit depth. Cloud-contaminated image. Output with a QSE circuit with depth *n* = 1. Output with a QSE circuit with depth *n* = 2. Output with a QSE circuit with depth *n* = 4. Output with a QSE circuit with depth *n* = 6. Corresponding cloud-free ground-truth image. QSE, quantum squeeze-and-excitation.

Compared to conventional baselines, the QSE-enhanced U-Net produces reconstructions with improved texture continuity, distinct structural boundaries, and lower artifact susceptibility. Although residual cloud artifacts are reduced, fine features are recreated more accurately. The two-layer QSE architecture provides the most perceptually coherent results, as verified by visual examination across different quantum depths. Deeper circuits usually produce oversmoothed outputs and suppress sensitive textures because of increasing non-linear mixing of channel representations. While modest mixing enhances contextual feature recalibration, excessive alterations may yield smoother reconstructions by suppressing high-frequency activations corresponding to fine textures and edges. Figure 4 shows representative qualitative assessments between ground-truth images, cloudy inputs, and reconstructed outputs.

### 3.11 Cross-dataset transfer evaluation on EuroSAT

**Figure 5:**
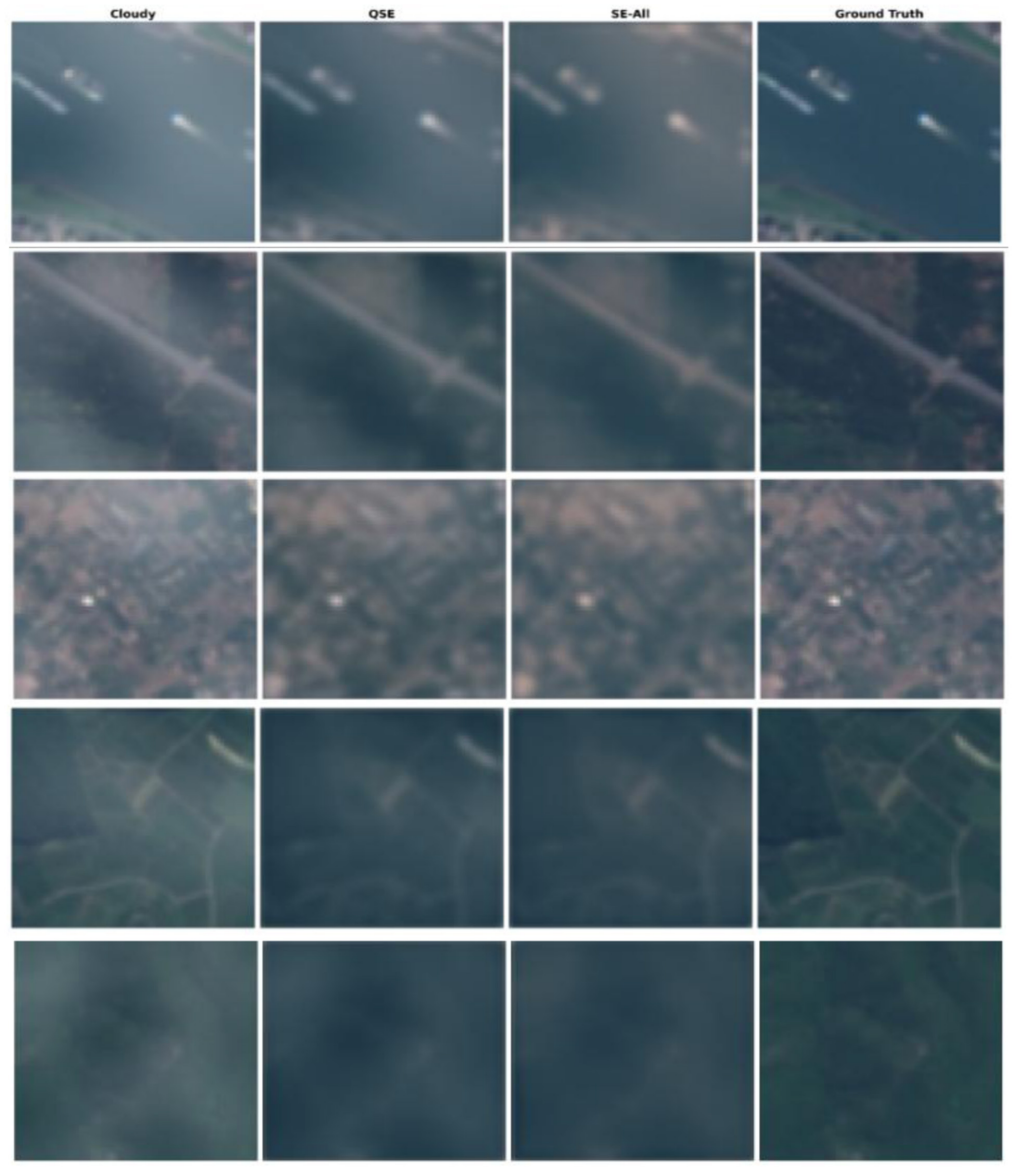
Qualitative cross-dataset transfer results on noise-augmented EuroSAT (herbaceous vegetation).

To evaluate cross-dataset transfer behavior, noise-augmented EuroSAT images are used to refine the proposed QSE-UNet and the previous SE-All architecture, which are then evaluated on the EuroSAT test split. The quantitative results, summarized in Table 9, show that QSE-based decoding yields a higher SSIM (0.9300 ± 0.0196) with less variance than SE-All (0.9144 ± 0.0319), indicating better structure preservation and more stable reconstruction under various conditions. In contrast to the RICE1 dataset, Figure 5 shows that the qualitative analysis reveals smoother reconstructions, even though both PSNR and SSIM values demonstrate strong quantitative performance on EuroSAT. This apparent discrepancy arises from the fact that PSNR and SSIM capture global fidelity and structural alignment rather than being particularly sensitive to high-frequency edge sharpness.

**Table 9:** Cross-dataset transfer performance on the EuroSAT dataset after fine-tuning on synthetically cloud-degraded images, comparing the proposed QSE-UNet with the prior SE-All model using PSNR and SSIM.

Model	PSNR (dB)	SSIM
SE-UNet (previous work)	28.83 ± 2.41	0.9144 ± 0.0319
QSE-UNet (proposed)	28.52 ± 2.22	0.9300 ± 0.0196

PSNR, peak signal-to-noise ratio; QSE, quantum squeeze-and-excitation; SE, squeeze-and-excitation; SSIM, structural similarity index measure.

Softer edges and partial cloud attenuation result from the model's conservative denoising technique, which prioritizes structural integrity over aggressive texture recovery under simulated atmospheric degradation. Importantly, QSE-UNet consistently increases SSIM and decreases variance despite the apparent smoothing, indicating improved robustness and stability under cross-dataset transfer.

The proposed QSE-enhanced time-conditioned U-Net achieves competitive results compared with conventional baselines in both quantitative and qualitative evaluations. Ablation studies demonstrate that decoder-level integration with moderate quantum circuit depth achieves the best trade-off between computational efficiency and reconstruction quality. These results are intended to show cross-dataset transfer behavior and validate the effectiveness of quantum-inspired channel attention for diffusion-style remote sensing image restoration, rather than establishing comprehensive performance on EuroSAT (He et al., 2025; Kerger and Miyazaki, 2023).

## 4 Limitations

While the proposed QSE-enhanced time-conditioned U-Net demonstrates consistent improvements over classical baselines, several limitations should be acknowledged.

First, all quantum components in this study are implemented using simulated quantum circuits running on classical hardware. Although this allows end-to-end differentiable training and controlled experimentation, it does not capture hardware-specific constraints such as noise, decoherence, and limited qubit connectivity present in near-term quantum devices. Consequently, the reported results reflect the potential of quantum-inspired attention mechanisms rather than performance on actual quantum hardware.

Second, cross-dataset evaluation under synthetic domain shift is conducted using synthetically generated Perlin noise-based cloud occlusions. While Perlin noise provides a controlled and spatially correlated approximation of cloud structures, it does not fully model the physical complexity and variability of real atmospheric cloud formations. As a result, the cross-dataset evaluation under synthetic domain shift should be interpreted as a stress test rather than a comprehensive assessment of real-world generalization.

Third, the additional non-linear transformations and entanglement operations produced by increasing circuit depth may interfere with optimization and increase gradient sensitivity during training. Excessive non-linear transformations may overmix intermediate channel representations and attenuate high-frequency spatial information necessary for effective restoration.

Finally, the proposed framework is evaluated on single-date optical satellite images and does not incorporate multi-temporal or multi-sensor information. Many operational cloud-removal systems leverage temporal redundancy or auxiliary modalities such as SAR imagery (Yadav et al., 2024; Dai et al., 2020). Integrating such information could further improve robustness, particularly in scenarios with dense or persistent cloud cover.

Finally, the computational overhead introduced by quantum circuit simulation increases training time compared to purely classical architectures. Although the parameter count remains comparable to classical SE-based models, scalability to higher resolutions or larger datasets may require additional optimization strategies.

## 5 Conclusion

This research proposes a hybrid quantum-classical framework for cloud removal in optical satellite data by incorporating QSE modules into a time-conditioned U-Net trained with a diffusion-inspired objective. The suggested method incorporates quantum-inspired channel attention as a structured non-linear feature recalibration mechanism for low-level image restoration.

Comprehensive testing on the RICE1 dataset demonstrates that the QSE-enhanced model consistently outperforms conventional U-Net and SE-U-Net baselines in terms of PSNR and SSIM while retaining stable training behavior. Ablation studies show that decoder-level integration of QSE modules with a shallow quantum circuit depth provides optimal reconstruction performance, emphasizing the significance of controlled quantum complexity and strategic placement. The qualitative findings also indicate higher-quality texture recovery and fewer cloud artifacts.

Additionally, the proposed method shows better structural preservation during cross-dataset transfer, indicating improved generalization behavior compared to classical attention mechanisms, based on controlled robustness evaluation under domain shift after fine-tuning on artificially generated cloud-like occlusions. Taken together, this research represents one of the earliest applications of quantum-inspired channel attention for diffusion-style remote sensing image restoration.

Further research could broaden the proposed framework to incorporate multi-modal SAR-optical fusion scenarios, physically grounded cloud modeling, and evaluation on larger remote sensing datasets exhibiting more intricate atmospheric variability. The practical use of hybrid quantum-classical restoration frameworks for remote sensing image reconstruction can also be enhanced further by incorporating scalable variational quantum circuit architectures.

## Data Availability

The datasets used in this study are publicly available and can be accessed at: https://github.com/BUPTLdy/RICE_DATASET.
